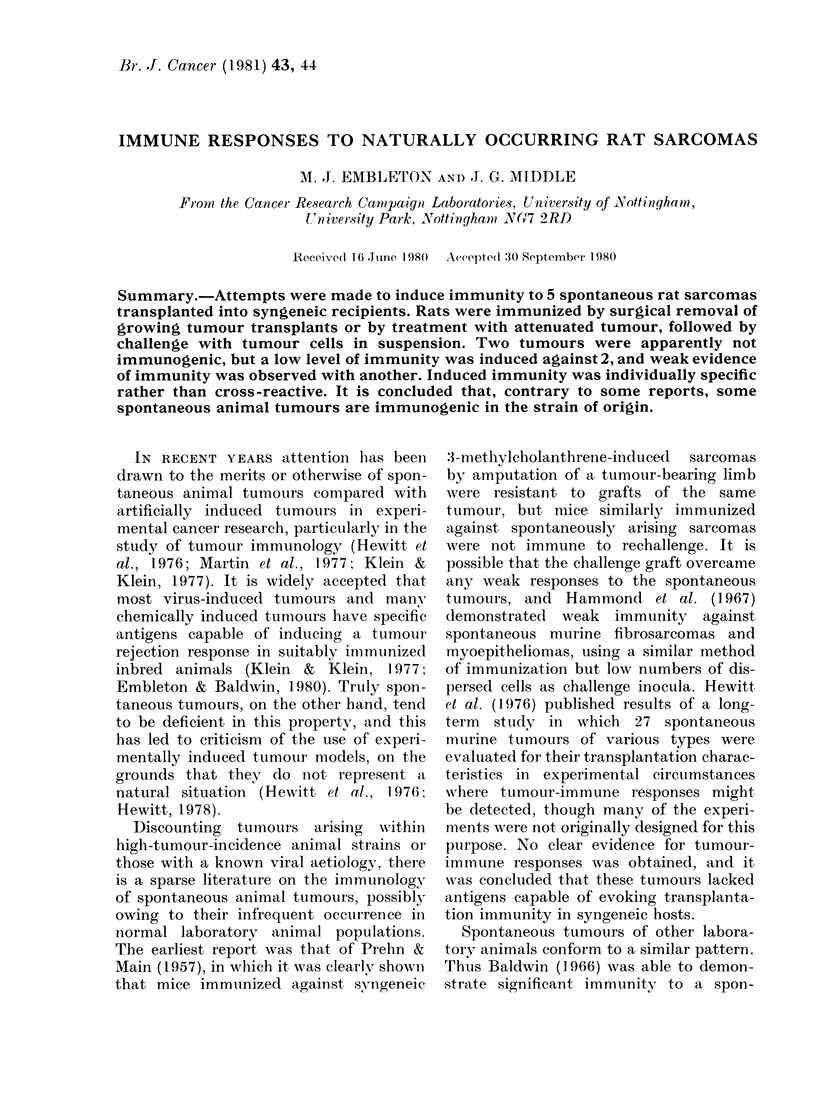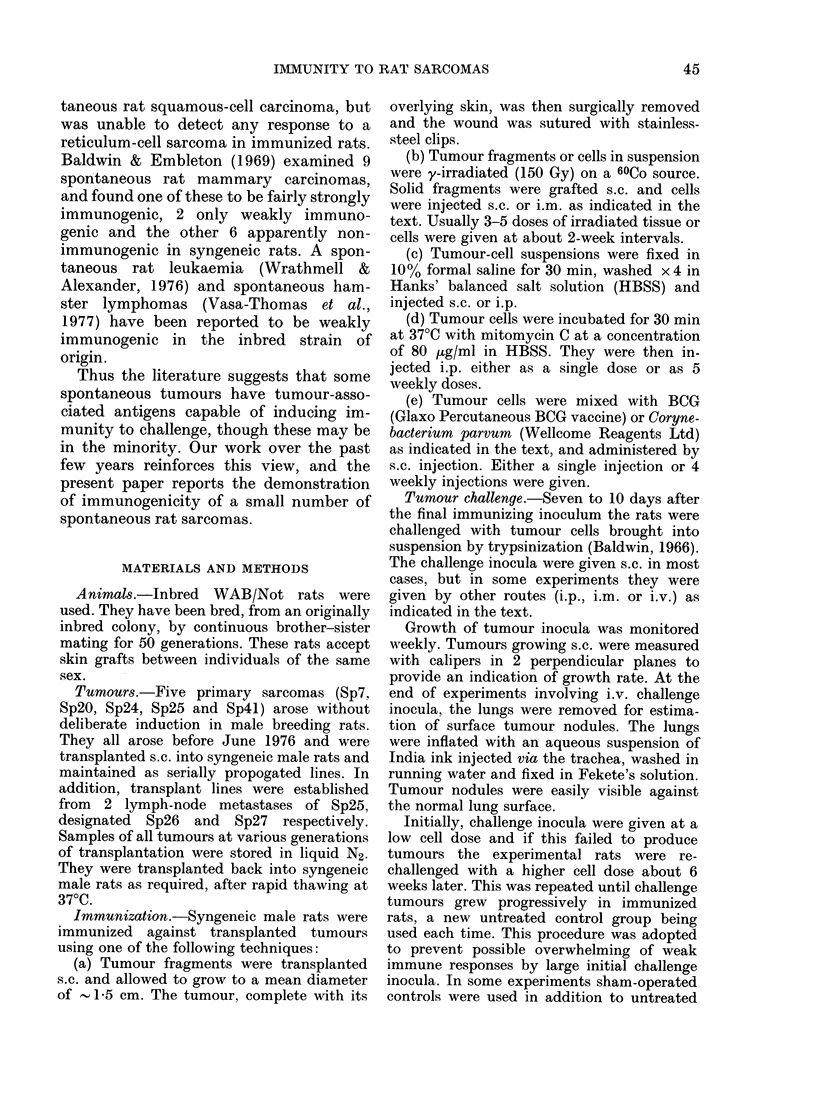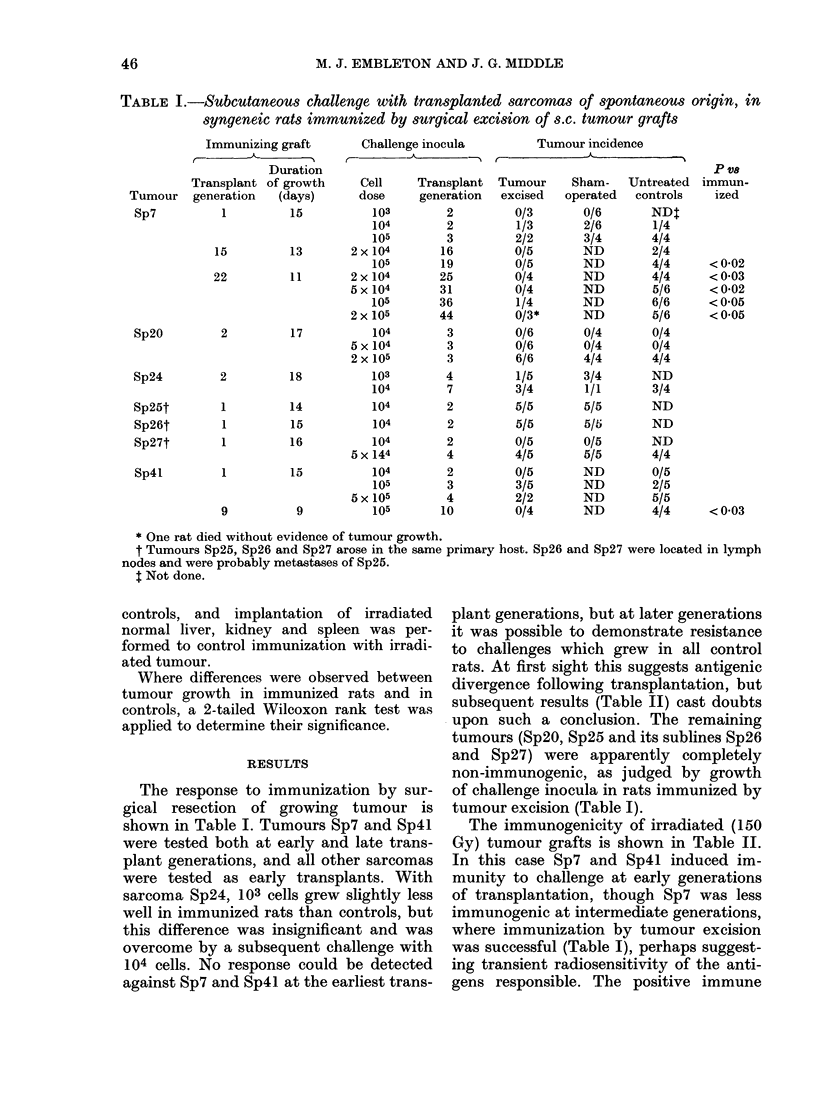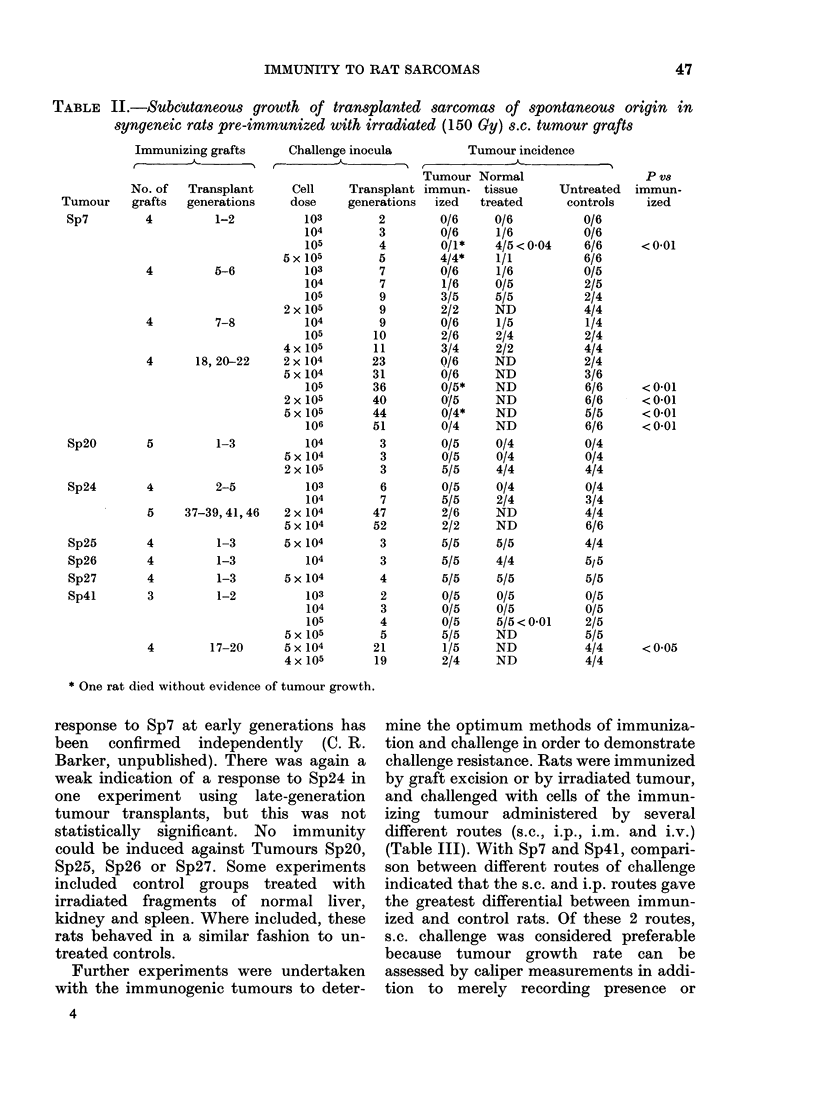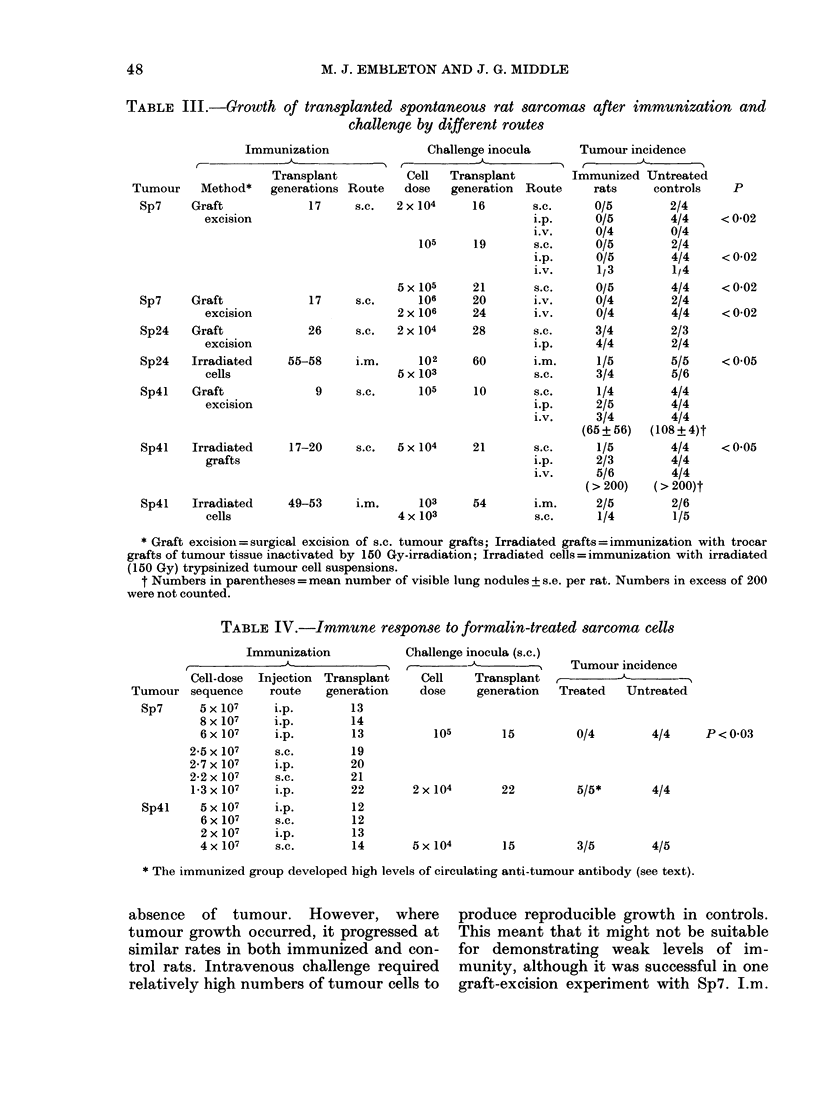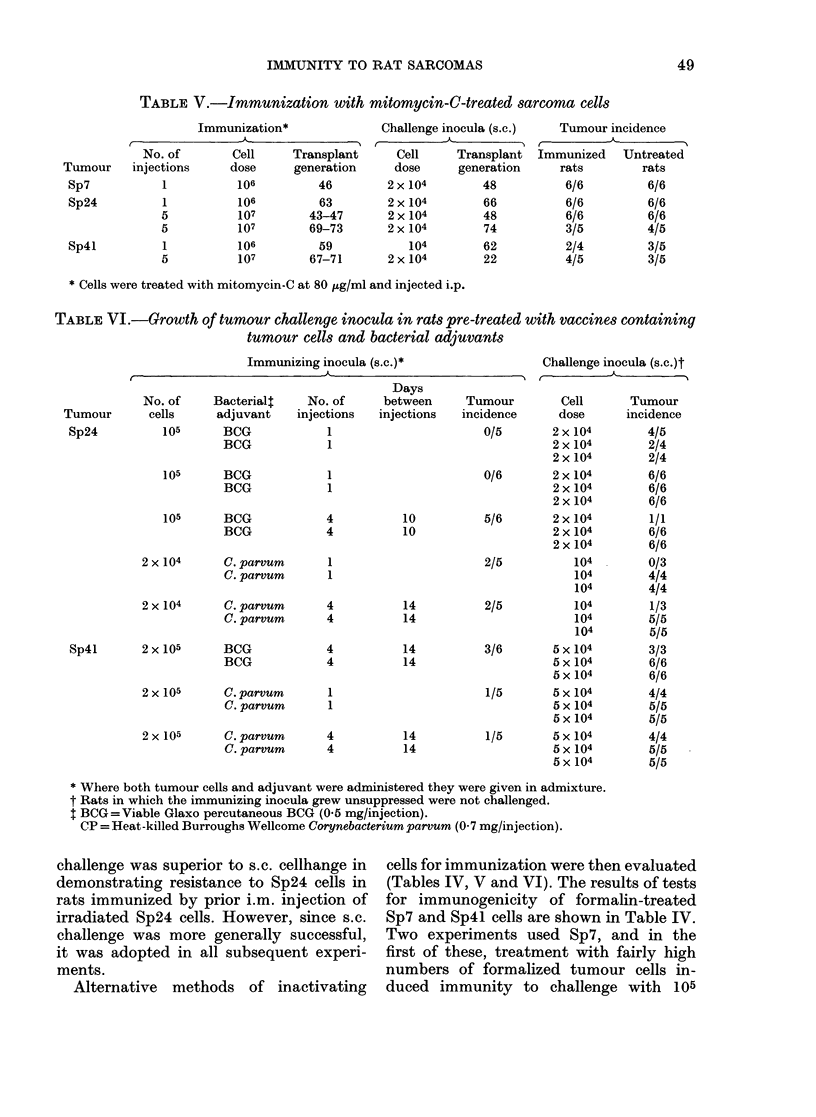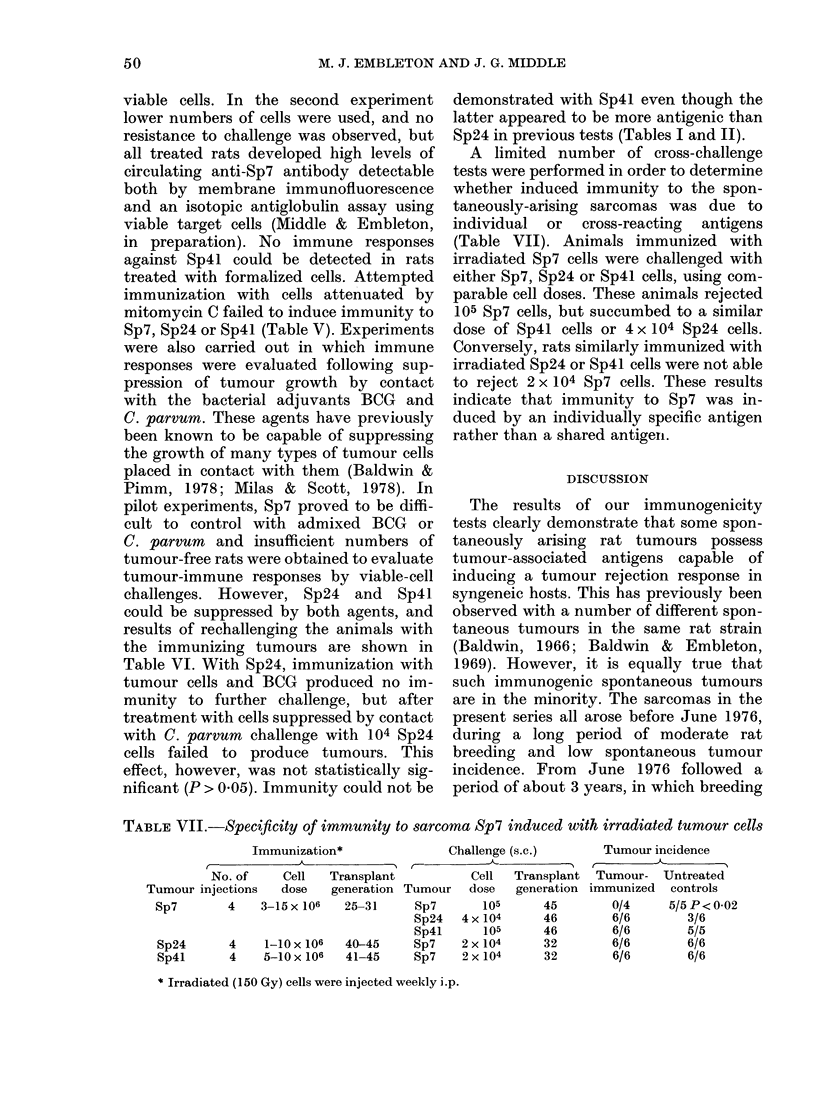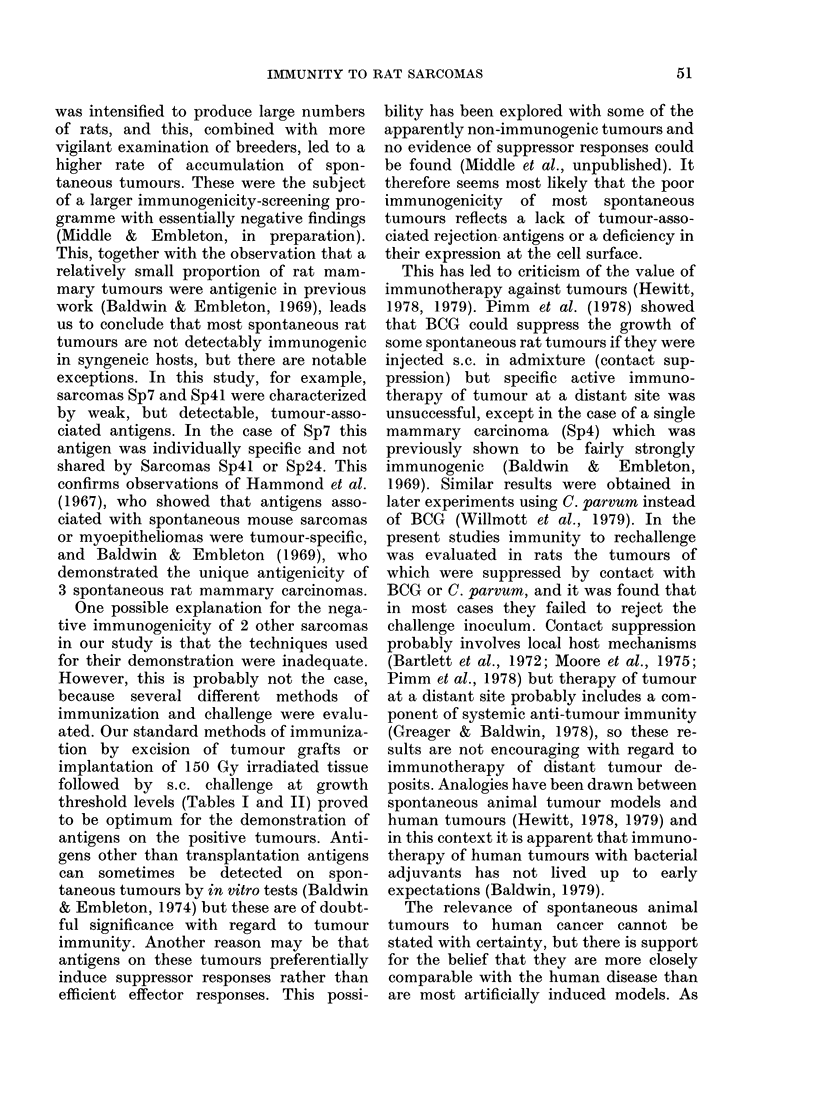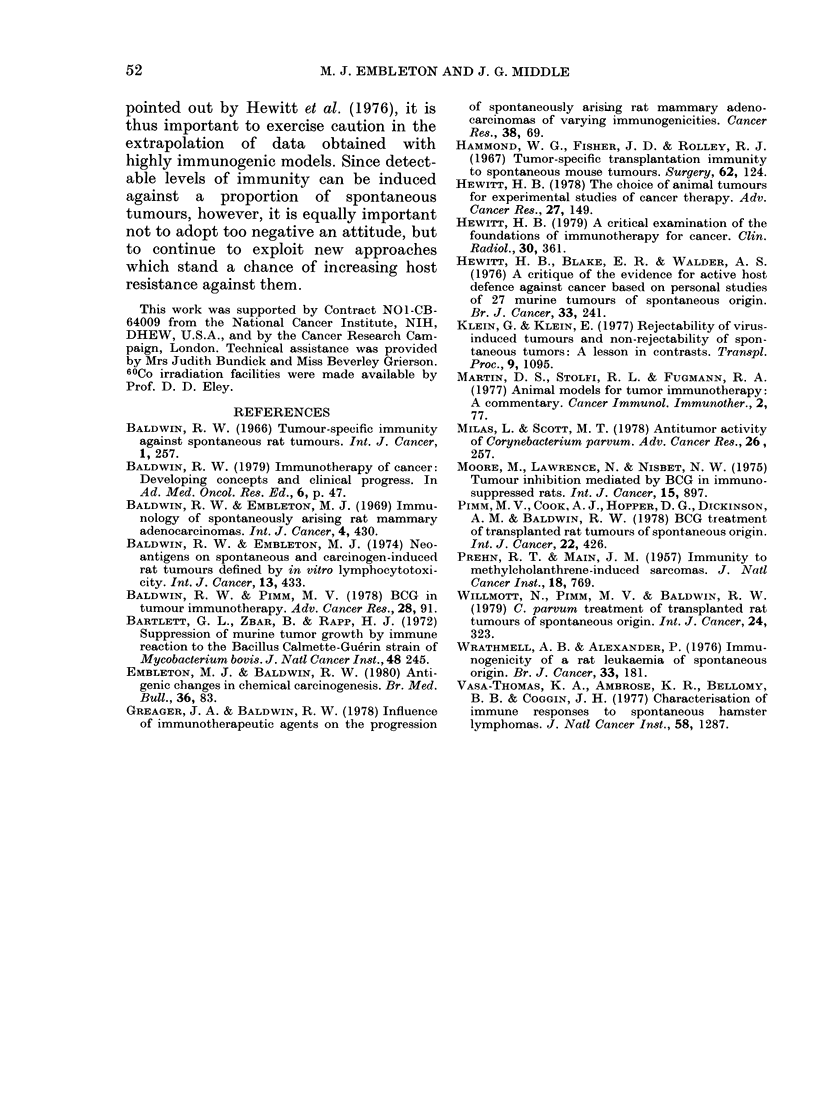# Immune responses to naturally occurring rat sarcomas.

**DOI:** 10.1038/bjc.1981.6

**Published:** 1981-01

**Authors:** M. J. Embleton, J. G. Middle

## Abstract

Attempts were made to induce immunity to 5 spontaneous rat sarcomas transplanted into syngeneic recipients. Rats were immunized by surgical removal of growing tumour transplants or by treatment with attenuated tumour, followed by challenge with tumour cells in suspension. Two tumours wee apparently not immunogenic, but a low level of immunity was induced against 2, and weak evidence of immunity was observed with another. Induced immunity was individually specific rather than cross-reactive. It is concluded that, contrary to some reports, some spontaneous animal tumours are immunogenic in the strain of origin.


*Br. J. Cancer* (1981) **43**, 44

### IMMUNE RESPONSES TO NATURALLY OCCURRING RAT SARCOMAS

M. J. EMBLETON AND J. G. MIDDLE

*From the Cancer Research Campaign Laboratories, University of Nottingham, University Park, Nottingham NG7 2RD*

Received 16 June 1980 Accepted 30 September 1980

**Summary.—Attempts were made to induce immunity to 5 spontaneous rat sarcomas transplanted into syngeneic recipients. Rats were immunized by surgical removal of growing tumour transplants or by treatment with attenuated tumour, followed by challenge with tumour cells in suspension. Two tumours were apparently not immunogenic, but a low level of immunity was induced against 2, and weak evidence of immunity was observed with another. Induced immunity was individually specific rather than cross-reactive. It is concluded that, contrary to some reports, some spontaneous animal tumours are immunogenic in the strain of origin.**

IN RECENT YEARS attention has been drawn to the merits or otherwise of spontaneous animal tumours compared with artificially induced tumours in experimental cancer research, particularly in the study of tumour immunology (Hewitt *et al.*, 1976; Martin *et al.*, 1977; Klein & Klein, 1977). It is widely accepted that most virus-induced tumours and many chemically induced tumours have specific antigens capable of inducing a tumour rejection response in suitably immunized inbred animals (Klein & Klein, 1977; Embleton & Baldwin, 1980). Truly spontaneous tumours, on the other hand, tend to be deficient in this property, and this has led to criticism of the use of experimentally induced tumour models, on the grounds that they do not represent a natural situation (Hewitt *et al.*, 1976; Hewitt, 1978).

Discounting tumours arising within high-tumour-incidence animal strains or those with a known viral aetiology, there is a sparse literature on the immunology of spontaneous animal tumours, possibly owing to their infrequent occurrence in normal laboratory animal populations. The earliest report was that of Prehn & Main (1957), in which it was clearly shown that mice immunized against syngeneic 3-methylcholanthrene-induced sarcomas by amputation of a tumour-bearing limb were resistant to grafts of the same tumour, but mice similarly immunized against spontaneously arising sarcomas were not immune to rechallenge. It is possible that the challenge graft overcame any weak responses to the spontaneous tumours, and Hammond *et al.* (1967) demonstrated weak immunity against spontaneous murine fibrosarcomas and myoepitheliomas, using a similar method of immunization but low numbers of dispersed cells as challenge inocula. Hewitt *et al.* (1976) published results of a long-term study in which 27 spontaneous murine tumours of various types were evaluated for their transplantation characteristics in experimental circumstances where tumour-immune responses might be detected, though many of the experiments were not originally designed for this purpose. No clear evidence for tumour-immune responses was obtained, and it was concluded that these tumours lacked antigens capable of evoking transplantation immunity in syngeneic hosts.

Spontaneous tumours of other laboratory animals conform to a similar pattern. Thus Baldwin (1966) was able to demonstrate significant immunity to a spontaneous rat squamous-cell carcinoma, but was unable to detect any response to a reticulum-cell sarcoma in immunized rats. Baldwin & Embleton (1969) examined 9 spontaneous rat mammary carcinomas, and found one of these to be fairly strongly immunogenic, 2 only weakly immunogenic and the other 6 apparently non-immunogenic in syngeneic rats. A spontaneous rat leukaemia (Wrathmell & Alexander, 1976) and spontaneous hamster lymphomas (Vasa-Thomas *et al.*, 1977) have been reported to be weakly immunogenic in the inbred strain of origin.

Thus the literature suggests that some spontaneous tumours have tumour-associated antigens capable of inducing immunity to challenge, though these may be in the minority. Our work over the past few years reinforces this view, and the present paper reports the demonstration of immunogenicity of a small number of spontaneous rat sarcomas.

### MATERIALS AND METHODS

*Animals.*—Inbred WAB/Not rats were used. They have been bred, from an originally inbred colony, by continuous brother–sister mating for 50 generations. These rats accept skin grafts between individuals of the same sex.

*Tumours.*—Five primary sarcomas (Sp7, Sp20, Sp24, Sp25 and Sp41) arose without deliberate induction in male breeding rats. They all arose before June 1976 and were transplanted s.c. into syngeneic male rats and maintained as serially propogated lines. In addition, transplant lines were established from 2 lymph-node metastases of Sp25, designated Sp26 and Sp27 respectively. Samples of all tumours at various generations of transplantation were stored in liquid $N_2$. They were transplanted back into syngeneic male rats as required, after rapid thawing at 37°C.

*Immunization.*—Syngeneic male rats were immunized against transplanted tumours using one of the following techniques:

(a) Tumour fragments were transplanted s.c. and allowed to grow to a mean diameter of ~1·5 cm. The tumour, complete with its overlying skin, was then surgically removed and the wound was sutured with stainless-steel clips.

(b) Tumour fragments or cells in suspension were γ-irradiated (150 Gy) on a $^{60}Co$ source. Solid fragments were grafted s.c. and cells were injected s.c. or i.m. as indicated in the text. Usually 3–5 doses of irradiated tissue or cells were given at about 2-week intervals.

(c) Tumour-cell suspensions were fixed in 10% formal saline for 30 min, washed ×4 in Hanks' balanced salt solution (HBSS) and injected s.c. or i.p.

(d) Tumour cells were incubated for 30 min at 37°C with mitomycin C at a concentration of 80 μg/ml in HBSS. They were then injected i.p. either as a single dose or as 5 weekly doses.

(e) Tumour cells were mixed with BCG (Glaxo Percutaneous BCG vaccine) or *Corynebacterium parvum* (Wellcome Reagents Ltd) as indicated in the text, and administered by s.c. injection. Either a single injection or 4 weekly injections were given.

*Tumour challenge.*—Seven to 10 days after the final immunizing inoculum the rats were challenged with tumour cells brought into suspension by trypsinization (Baldwin, 1966). The challenge inocula were given s.c. in most cases, but in some experiments they were given by other routes (i.p., i.m. or i.v.) as indicated in the text.

Growth of tumour inocula was monitored weekly. Tumours growing s.c. were measured with calipers in 2 perpendicular planes to provide an indication of growth rate. At the end of experiments involving i.v. challenge inocula, the lungs were removed for estimation of surface tumour nodules. The lungs were inflated with an aqueous suspension of India ink injected *via* the trachea, washed in running water and fixed in Fekete's solution. Tumour nodules were easily visible against the normal lung surface.

Initially, challenge inocula were given at a low cell dose and if this failed to produce tumours the experimental rats were re-challenged with a higher cell dose about 6 weeks later. This was repeated until challenge tumours grew progressively in immunized rats, a new untreated control group being used each time. This procedure was adopted to prevent possible overwhelming of weak immune responses by large initial challenge inocula. In some experiments sham-operated controls were used in addition to untreated

TABLE I.—*Subcutaneous challenge with transplanted sarcomas of spontaneous origin, in syngeneic rats immunized by surgical excision of s.c. tumour grafts*

| | Immunizing graft | | Challenge inocula | | Tumour incidence | | | |
|---|---|---|---|---|---|---|---|---|
| Tumour | Transplant generation | Duration of growth (days) | Cell dose | Transplant generation | Tumour excised | Sham-operated | Untreated controls | *P vs* immunized |
| Sp7 | 1 | 15 | $10^3$ | 2 | 0/3 | 0/6 | ND‡ | |
| | | | $10^4$ | 2 | 1/3 | 2/6 | 1/4 | |
| | | | $10^5$ | 3 | 2/2 | 3/4 | 4/4 | |
| | 15 | 13 | $2\times10^4$ | 16 | 0/5 | ND | 2/4 | |
| | | | $10^5$ | 19 | 0/5 | ND | 4/4 | $<0{\cdot}02$ |
| | 22 | 11 | $2\times10^4$ | 25 | 0/4 | ND | 4/4 | $<0{\cdot}03$ |
| | | | $5\times10^4$ | 31 | 0/4 | ND | 5/6 | $<0{\cdot}02$ |
| | | | $10^5$ | 36 | 1/4 | ND | 6/6 | $<0{\cdot}05$ |
| | | | $2\times10^5$ | 44 | 0/3* | ND | 5/6 | $<0{\cdot}05$ |
| Sp20 | 2 | 17 | $10^4$ | 3 | 0/6 | 0/4 | 0/4 | |
| | | | $5\times10^4$ | 3 | 0/6 | 0/4 | 0/4 | |
| | | | $2\times10^5$ | 3 | 6/6 | 4/4 | 4/4 | |
| Sp24 | 2 | 18 | $10^3$ | 4 | 1/5 | 3/4 | ND | |
| | | | $10^4$ | 7 | 3/4 | 1/1 | 3/4 | |
| Sp25† | 1 | 14 | $10^4$ | 2 | 5/5 | 5/5 | ND | |
| Sp26† | 1 | 15 | $10^4$ | 2 | 5/5 | 5/5 | ND | |
| Sp27† | 1 | 16 | $10^4$ | 2 | 0/5 | 0/5 | ND | |
| | | | $5\times14^4$ | 4 | 4/5 | 5/5 | 4/4 | |
| Sp41 | 1 | 15 | $10^4$ | 2 | 0/5 | ND | 0/5 | |
| | | | $10^5$ | 3 | 3/5 | ND | 2/5 | |
| | | | $5\times10^5$ | 4 | 2/2 | ND | 5/5 | |
| | 9 | 9 | $10^5$ | 10 | 0/4 | ND | 4/4 | $<0{\cdot}03$ |

* One rat died without evidence of tumour growth.

† Tumours Sp25, Sp26 and Sp27 arose in the same primary host. Sp26 and Sp27 were located in lymph nodes and were probably metastases of Sp25.

‡ Not done.

controls, and implantation of irradiated normal liver, kidney and spleen was performed to control immunization with irradiated tumour.

Where differences were observed between tumour growth in immunized rats and in controls, a 2-tailed Wilcoxon rank test was applied to determine their significance.

### RESULTS

The response to immunization by surgical resection of growing tumour is shown in Table I. Tumours Sp7 and Sp41 were tested both at early and late transplant generations, and all other sarcomas were tested as early transplants. With sarcoma Sp24, $10^3$ cells grew slightly less well in immunized rats than controls, but this difference was insignificant and was overcome by a subsequent challenge with $10^4$ cells. No response could be detected against Sp7 and Sp41 at the earliest transplant generations, but at later generations it was possible to demonstrate resistance to challenges which grew in all control rats. At first sight this suggests antigenic divergence following transplantation, but subsequent results (Table II) cast doubts upon such a conclusion. The remaining tumours (Sp20, Sp25 and its sublines Sp26 and Sp27) were apparently completely non-immunogenic, as judged by growth of challenge inocula in rats immunized by tumour excision (Table I).

The immunogenicity of irradiated (150 Gy) tumour grafts is shown in Table II. In this case Sp7 and Sp41 induced immunity to challenge at early generations of transplantation, though Sp7 was less immunogenic at intermediate generations, where immunization by tumour excision was successful (Table I), perhaps suggesting transient radiosensitivity of the antigens responsible. The positive immune

TABLE II.—*Subcutaneous growth of transplanted sarcomas of spontaneous origin in syngeneic rats pre-immunized with irradiated* (150 *Gy*) *s.c. tumour grafts*

| | Immunizing grafts | | Challenge inocula | | Tumour incidence | | | |
|---|---|---|---|---|---|---|---|---|
| Tumour | No. of grafts | Transplant generations | Cell dose | Transplant generations | Tumour immunized | Normal tissue treated | Untreated controls | *P vs* immunized |
| Sp7 | 4 | 1–2 | $10^3$ | 2 | 0/6 | 0/6 | 0/6 | |
| | | | $10^4$ | 3 | 0/6 | 1/6 | 0/6 | |
| | | | $10^5$ | 4 | 0/1* | 4/5 < 0·04 | 6/6 | < 0·01 |
| | | | $5 \times 10^5$ | 5 | 4/4* | 1/1 | 6/6 | |
| | 4 | 5–6 | $10^3$ | 7 | 0/6 | 1/6 | 0/5 | |
| | | | $10^4$ | 7 | 1/6 | 0/5 | 2/5 | |
| | | | $10^5$ | 9 | 3/5 | 5/5 | 2/4 | |
| | | | $2 \times 10^5$ | 9 | 2/2 | ND | 4/4 | |
| | 4 | 7–8 | $10^4$ | 9 | 0/6 | 1/5 | 1/4 | |
| | | | $10^5$ | 10 | 2/6 | 2/4 | 2/4 | |
| | | | $4 \times 10^5$ | 11 | 3/4 | 2/2 | 4/4 | |
| | 4 | 18, 20–22 | $2 \times 10^4$ | 23 | 0/6 | ND | 2/4 | |
| | | | $5 \times 10^4$ | 31 | 0/6 | ND | 3/6 | |
| | | | $10^5$ | 36 | 0/5* | ND | 6/6 | < 0·01 |
| | | | $2 \times 10^5$ | 40 | 0/5 | ND | 6/6 | < 0·01 |
| | | | $5 \times 10^5$ | 44 | 0/4* | ND | 5/5 | < 0·01 |
| | | | $10^6$ | 51 | 0/4 | ND | 6/6 | < 0·01 |
| Sp20 | 5 | 1–3 | $10^4$ | 3 | 0/5 | 0/4 | 0/4 | |
| | | | $5 \times 10^4$ | 3 | 0/5 | 0/4 | 0/4 | |
| | | | $2 \times 10^5$ | 3 | 5/5 | 4/4 | 4/4 | |
| Sp24 | 4 | 2–5 | $10^3$ | 6 | 0/5 | 0/4 | 0/4 | |
| | | | $10^4$ | 7 | 5/5 | 2/4 | 3/4 | |
| | 5 | 37–39, 41, 46 | $2 \times 10^4$ | 47 | 2/6 | ND | 4/4 | |
| | | | $5 \times 10^4$ | 52 | 2/2 | ND | 6/6 | |
| Sp25 | 4 | 1–3 | $5 \times 10^4$ | 3 | 5/5 | 5/5 | 4/4 | |
| Sp26 | 4 | 1–3 | $10^4$ | 3 | 5/5 | 4/4 | 5/5 | |
| Sp27 | 4 | 1–3 | $5 \times 10^4$ | 4 | 5/5 | 5/5 | 5/5 | |
| Sp41 | 3 | 1–2 | $10^3$ | 2 | 0/5 | 0/5 | 0/5 | |
| | | | $10^4$ | 3 | 0/5 | 0/5 | 0/5 | |
| | | | $10^5$ | 4 | 0/5 | 5/5 < 0·01 | 2/5 | |
| | | | $5 \times 10^5$ | 5 | 5/5 | ND | 5/5 | |
| | 4 | 17–20 | $5 \times 10^4$ | 21 | 1/5 | ND | 4/4 | < 0·05 |
| | | | $4 \times 10^5$ | 19 | 2/4 | ND | 4/4 | |

* One rat died without evidence of tumour growth.

response to Sp7 at early generations has been confirmed independently (C. R. Barker, unpublished). There was again a weak indication of a response to Sp24 in one experiment using late-generation tumour transplants, but this was not statistically significant. No immunity could be induced against Tumours Sp20, Sp25, Sp26 or Sp27. Some experiments included control groups treated with irradiated fragments of normal liver, kidney and spleen. Where included, these rats behaved in a similar fashion to untreated controls.

Further experiments were undertaken with the immunogenic tumours to determine the optimum methods of immunization and challenge in order to demonstrate challenge resistance. Rats were immunized by graft excision or by irradiated tumour, and challenged with cells of the immunizing tumour administered by several different routes (s.c., i.p., i.m. and i.v.) (Table III). With Sp7 and Sp41, comparison between different routes of challenge indicated that the s.c. and i.p. routes gave the greatest differential between immunized and control rats. Of these 2 routes, s.c. challenge was considered preferable because tumour growth rate can be assessed by caliper measurements in addition to merely recording presence or

TABLE III.—*Growth of transplanted spontaneous rat sarcomas after immunization and challenge by different routes*

| | Immunization | | | Challenge inocula | | | Tumour incidence | | |
|---|---|---|---|---|---|---|---|---|---|
| Tumour | Method* | Transplant generations | Route | Cell dose | Transplant generation | Route | Immunized rats | Untreated controls | *P* |
| Sp7 | Graft excision | 17 | s.c. | $2\times10^4$ | 16 | s.c. | 0/5 | 2/4 | |
| | | | | | | i.p. | 0/5 | 4/4 | $<0\cdot02$ |
| | | | | | | i.v. | 0/4 | 0/4 | |
| | | | | $10^5$ | 19 | s.c. | 0/5 | 2/4 | |
| | | | | | | i.p. | 0/5 | 4/4 | $<0\cdot02$ |
| | | | | | | i.v. | 1/3 | 1/4 | |
| | | | | $5\times10^5$ | 21 | s.c. | 0/5 | 4/4 | $<0\cdot02$ |
| Sp7 | Graft excision | 17 | s.c. | $10^6$ | 20 | i.v. | 0/4 | 2/4 | |
| | | | | $2\times10^6$ | 24 | i.v. | 0/4 | 4/4 | $<0\cdot02$ |
| Sp24 | Graft excision | 26 | s.c. | $2\times10^4$ | 28 | s.c. | 3/4 | 2/3 | |
| | | | | | | i.p. | 4/4 | 2/4 | |
| Sp24 | Irradiated cells | 55–58 | i.m. | $10^2$ | 60 | i.m. | 1/5 | 5/5 | $<0\cdot05$ |
| | | | | $5\times10^3$ | | s.c. | 3/4 | 5/6 | |
| Sp41 | Graft excision | 9 | s.c. | $10^5$ | 10 | s.c. | 1/4 | 4/4 | |
| | | | | | | i.p. | 2/5 | 4/4 | |
| | | | | | | i.v. | 3/4 (65 ± 56) | 4/4 (108 ± 4)† | |
| Sp41 | Irradiated grafts | 17–20 | s.c. | $5\times10^4$ | 21 | s.c. | 1/5 | 4/4 | $<0\cdot05$ |
| | | | | | | i.p. | 2/3 | 4/4 | |
| | | | | | | i.v. | 5/6 (> 200) | 4/4 (> 200)† | |
| Sp41 | Irradiated cells | 49–53 | i.m. | $10^3$ | 54 | i.m. | 2/5 | 2/6 | |
| | | | | $4\times10^3$ | | s.c. | 1/4 | 1/5 | |

* Graft excision = surgical excision of s.c. tumour grafts; Irradiated grafts = immunization with trocar grafts of tumour tissue inactivated by 150 Gy-irradiation; Irradiated cells = immunization with irradiated (150 Gy) trypsinized tumour cell suspensions.

† Numbers in parentheses = mean number of visible lung nodules ± s.e. per rat. Numbers in excess of 200 were not counted.

TABLE IV.—*Immune response to formalin-treated sarcoma cells*

| | Immunization | | | Challenge inocula (s.c.) | | Tumour incidence | | |
|---|---|---|---|---|---|---|---|---|
| Tumour | Cell-dose sequence | Injection route | Transplant generation | Cell dose | Transplant generation | Treated | Untreated | |
| Sp7 | $5\times10^7$ | i.p. | 13 | | | | | |
| | $8\times10^7$ | i.p. | 14 | | | | | |
| | $6\times10^7$ | i.p. | 13 | $10^5$ | 15 | 0/4 | 4/4 | $P<0\cdot03$ |
| | $2\cdot5\times10^7$ | s.c. | 19 | | | | | |
| | $2\cdot7\times10^7$ | i.p. | 20 | | | | | |
| | $2\cdot2\times10^7$ | s.c. | 21 | | | | | |
| | $1\cdot3\times10^7$ | i.p. | 22 | $2\times10^4$ | 22 | 5/5* | 4/4 | |
| Sp41 | $5\times10^7$ | i.p. | 12 | | | | | |
| | $6\times10^7$ | s.c. | 12 | | | | | |
| | $2\times10^7$ | i.p. | 13 | | | | | |
| | $4\times10^7$ | s.c. | 14 | $5\times10^4$ | 15 | 3/5 | 4/5 | |

* The immunized group developed high levels of circulating anti-tumour antibody (see text).

absence of tumour. However, where tumour growth occurred, it progressed at similar rates in both immunized and control rats. Intravenous challenge required relatively high numbers of tumour cells to produce reproducible growth in controls. This meant that it might not be suitable for demonstrating weak levels of immunity, although it was successful in one graft-excision experiment with Sp7. I.m.

TABLE V.—*Immunization with mitomycin-C-treated sarcoma cells*

| | Immunization* | | | Challenge inocula (s.c.) | | Tumour incidence | |
|---|---|---|---|---|---|---|---|
| Tumour | No. of injections | Cell dose | Transplant generation | Cell dose | Transplant generation | Immunized rats | Untreated rats |
| Sp7 | 1 | $10^6$ | 46 | $2\times10^4$ | 48 | 6/6 | 6/6 |
| Sp24 | 1 | $10^6$ | 63 | $2\times10^4$ | 66 | 6/6 | 6/6 |
| | 5 | $10^7$ | 43–47 | $2\times10^4$ | 48 | 6/6 | 6/6 |
| | 5 | $10^7$ | 69–73 | $2\times10^4$ | 74 | 3/5 | 4/5 |
| Sp41 | 1 | $10^6$ | 59 | $10^4$ | 62 | 2/4 | 3/5 |
| | 5 | $10^7$ | 67–71 | $2\times10^4$ | 22 | 4/5 | 3/5 |

* Cells were treated with mitomycin-C at 80 μg/ml and injected i.p.

TABLE VI.—*Growth of tumour challenge inocula in rats pre-treated with vaccines containing tumour cells and bacterial adjuvants*

| | Immunizing inocula (s.c.)* | | | | | Challenge inocula (s.c.)† | |
|---|---|---|---|---|---|---|---|
| Tumour | No. of cells | Bacterial‡ adjuvant | No. of injections | Days between injections | Tumour incidence | Cell dose | Tumour incidence |
| Sp24 | $10^5$ | BCG | 1 | | 0/5 | $2\times10^4$ | 4/5 |
| | | BCG | 1 | | | $2\times10^4$ | 2/4 |
| | | | | | | $2\times10^4$ | 2/4 |
| | $10^5$ | BCG | 1 | | 0/6 | $2\times10^4$ | 6/6 |
| | | BCG | 1 | | | $2\times10^4$ | 6/6 |
| | | | | | | $2\times10^4$ | 6/6 |
| | $10^5$ | BCG | 4 | 10 | 5/6 | $2\times10^4$ | 1/1 |
| | | BCG | 4 | 10 | | $2\times10^4$ | 6/6 |
| | | | | | | $2\times10^4$ | 6/6 |
| | $2\times10^4$ | *C. parvum* | 1 | | 2/5 | $10^4$ | 0/3 |
| | | *C. parvum* | 1 | | | $10^4$ | 4/4 |
| | | | | | | $10^4$ | 4/4 |
| | $2\times10^4$ | *C. parvum* | 4 | 14 | 2/5 | $10^4$ | 1/3 |
| | | *C. parvum* | 4 | 14 | | $10^4$ | 5/5 |
| | | | | | | $10^4$ | 5/5 |
| Sp41 | $2\times10^5$ | BCG | 4 | 14 | 3/6 | $5\times10^4$ | 3/3 |
| | | BCG | 4 | 14 | | $5\times10^4$ | 6/6 |
| | | | | | | $5\times10^4$ | 6/6 |
| | $2\times10^5$ | *C. parvum* | 1 | | 1/5 | $5\times10^4$ | 4/4 |
| | | *C. parvum* | 1 | | | $5\times10^4$ | 5/5 |
| | | | | | | $5\times10^4$ | 5/5 |
| | $2\times10^5$ | *C. parvum* | 4 | 14 | 1/5 | $5\times10^4$ | 4/4 |
| | | *C. parvum* | 4 | 14 | | $5\times10^4$ | 5/5 |
| | | | | | | $5\times10^4$ | 5/5 |

* Where both tumour cells and adjuvant were administered they were given in admixture.
† Rats in which the immunizing inocula grew unsuppressed were not challenged.
‡ BCG = Viable Glaxo percutaneous BCG (0·5 mg/injection).
CP = Heat-killed Burroughs Wellcome *Corynebacterium parvum* (0·7 mg/injection).

challenge was superior to s.c. cellhange in demonstrating resistance to Sp24 cells in rats immunized by prior i.m. injection of irradiated Sp24 cells. However, since s.c. challenge was more generally successful, it was adopted in all subsequent experiments.

Alternative methods of inactivating cells for immunization were then evaluated (Tables IV, V and VI). The results of tests for immunogenicity of formalin-treated Sp7 and Sp41 cells are shown in Table IV. Two experiments used Sp7, and in the first of these, treatment with fairly high numbers of formalized tumour cells induced immunity to challenge with $10^5$

viable cells. In the second experiment lower numbers of cells were used, and no resistance to challenge was observed, but all treated rats developed high levels of circulating anti-Sp7 antibody detectable both by membrane immunofluorescence and an isotopic antiglobulin assay using viable target cells (Middle & Embleton, in preparation). No immune responses against Sp41 could be detected in rats treated with formalized cells. Attempted immunization with cells attenuated by mitomycin C failed to induce immunity to Sp7, Sp24 or Sp41 (Table V). Experiments were also carried out in which immune responses were evaluated following suppression of tumour growth by contact with the bacterial adjuvants BCG and *C. parvum*. These agents have previously been known to be capable of suppressing the growth of many types of tumour cells placed in contact with them (Baldwin & Pimm, 1978; Milas & Scott, 1978). In pilot experiments, Sp7 proved to be difficult to control with admixed BCG or *C. parvum* and insufficient numbers of tumour-free rats were obtained to evaluate tumour-immune responses by viable-cell challenges. However, Sp24 and Sp41 could be suppressed by both agents, and results of rechallenging the animals with the immunizing tumours are shown in Table VI. With Sp24, immunization with tumour cells and BCG produced no immunity to further challenge, but after treatment with cells suppressed by contact with *C. parvum* challenge with $10^4$ Sp24 cells failed to produce tumours. This effect, however, was not statistically significant ($P > 0{\cdot}05$). Immunity could not be demonstrated with Sp41 even though the latter appeared to be more antigenic than Sp24 in previous tests (Tables I and II).

A limited number of cross-challenge tests were performed in order to determine whether induced immunity to the spontaneously-arising sarcomas was due to individual or cross-reacting antigens (Table VII). Animals immunized with irradiated Sp7 cells were challenged with either Sp7, Sp24 or Sp41 cells, using comparable cell doses. These animals rejected $10^5$ Sp7 cells, but succumbed to a similar dose of Sp41 cells or $4 \times 10^4$ Sp24 cells. Conversely, rats similarly immunized with irradiated Sp24 or Sp41 cells were not able to reject $2 \times 10^4$ Sp7 cells. These results indicate that immunity to Sp7 was induced by an individually specific antigen rather than a shared antigen.

### DISCUSSION

The results of our immunogenicity tests clearly demonstrate that some spontaneously arising rat tumours possess tumour-associated antigens capable of inducing a tumour rejection response in syngeneic hosts. This has previously been observed with a number of different spontaneous tumours in the same rat strain (Baldwin, 1966; Baldwin & Embleton, 1969). However, it is equally true that such immunogenic spontaneous tumours are in the minority. The sarcomas in the present series all arose before June 1976, during a long period of moderate rat breeding and low spontaneous tumour incidence. From June 1976 followed a period of about 3 years, in which breeding

TABLE VII.—*Specificity of immunity to sarcoma Sp7 induced with irradiated tumour cells*

| Immunization* | | | | Challenge (s.c.) | | | Tumour incidence | |
|---|---|---|---|---|---|---|---|---|
| Tumour | No. of injections | Cell dose | Transplant generation | Tumour | Cell dose | Transplant generation | Tumour-immunized | Untreated controls |
| Sp7 | 4 | $3–15 \times 10^6$ | 25–31 | Sp7 | $10^5$ | 45 | 0/4 | 5/5 $P < 0{\cdot}02$ |
| | | | | Sp24 | $4 \times 10^4$ | 46 | 6/6 | 3/6 |
| | | | | Sp41 | $10^5$ | 46 | 6/6 | 5/5 |
| Sp24 | 4 | $1–10 \times 10^6$ | 40–45 | Sp7 | $2 \times 10^4$ | 32 | 6/6 | 6/6 |
| Sp41 | 4 | $5–10 \times 10^6$ | 41–45 | Sp7 | $2 \times 10^4$ | 32 | 6/6 | 6/6 |

* Irradiated (150 Gy) cells were injected weekly i.p.

was intensified to produce large numbers of rats, and this, combined with more vigilant examination of breeders, led to a higher rate of accumulation of spontaneous tumours. These were the subject of a larger immunogenicity-screening programme with essentially negative findings (Middle & Embleton, in preparation). This, together with the observation that a relatively small proportion of rat mammary tumours were antigenic in previous work (Baldwin & Embleton, 1969), leads us to conclude that most spontaneous rat tumours are not detectably immunogenic in syngeneic hosts, but there are notable exceptions. In this study, for example, sarcomas Sp7 and Sp41 were characterized by weak, but detectable, tumour-associated antigens. In the case of Sp7 this antigen was individually specific and not shared by Sarcomas Sp41 or Sp24. This confirms observations of Hammond *et al.* (1967), who showed that antigens associated with spontaneous mouse sarcomas or myoepitheliomas were tumour-specific, and Baldwin & Embleton (1969), who demonstrated the unique antigenicity of 3 spontaneous rat mammary carcinomas.

One possible explanation for the negative immunogenicity of 2 other sarcomas in our study is that the techniques used for their demonstration were inadequate. However, this is probably not the case, because several different methods of immunization and challenge were evaluated. Our standard methods of immunization by excision of tumour grafts or implantation of 150 Gy irradiated tissue followed by s.c. challenge at growth threshold levels (Tables I and II) proved to be optimum for the demonstration of antigens on the positive tumours. Antigens other than transplantation antigens can sometimes be detected on spontaneous tumours by *in vitro* tests (Baldwin & Embleton, 1974) but these are of doubtful significance with regard to tumour immunity. Another reason may be that antigens on these tumours preferentially induce suppressor responses rather than efficient effector responses. This possibility has been explored with some of the apparently non-immunogenic tumours and no evidence of suppressor responses could be found (Middle *et al.*, unpublished). It therefore seems most likely that the poor immunogenicity of most spontaneous tumours reflects a lack of tumour-associated rejection antigens or a deficiency in their expression at the cell surface.

This has led to criticism of the value of immunotherapy against tumours (Hewitt, 1978, 1979). Pimm *et al.* (1978) showed that BCG could suppress the growth of some spontaneous rat tumours if they were injected s.c. in admixture (contact suppression) but specific active immunotherapy of tumour at a distant site was unsuccessful, except in the case of a single mammary carcinoma (Sp4) which was previously shown to be fairly strongly immunogenic (Baldwin & Embleton, 1969). Similar results were obtained in later experiments using *C. parvum* instead of BCG (Willmott *et al.*, 1979). In the present studies immunity to rechallenge was evaluated in rats the tumours of which were suppressed by contact with BCG or *C. parvum*, and it was found that in most cases they failed to reject the challenge inoculum. Contact suppression probably involves local host mechanisms (Bartlett *et al.*, 1972; Moore *et al.*, 1975; Pimm *et al.*, 1978) but therapy of tumour at a distant site probably includes a component of systemic anti-tumour immunity (Greager & Baldwin, 1978), so these results are not encouraging with regard to immunotherapy of distant tumour deposits. Analogies have been drawn between spontaneous animal tumour models and human tumours (Hewitt, 1978, 1979) and in this context it is apparent that immunotherapy of human tumours with bacterial adjuvants has not lived up to early expectations (Baldwin, 1979).

The relevance of spontaneous animal tumours to human cancer cannot be stated with certainty, but there is support for the belief that they are more closely comparable with the human disease than are most artificially induced models. As pointed out by Hewitt *et al.* (1976), it is thus important to exercise caution in the extrapolation of data obtained with highly immunogenic models. Since detectable levels of immunity can be induced against a proportion of spontaneous tumours, however, it is equally important not to adopt too negative an attitude, but to continue to exploit new approaches which stand a chance of increasing host resistance against them.

This work was supported by Contract NO1-CB-64009 from the National Cancer Institute, NIH, DHEW, U.S.A., and by the Cancer Research Campaign, London. Technical assistance was provided by Mrs Judith Bundick and Miss Beverley Grierson. $^{60}$Co irradiation facilities were made available by Prof. D. D. Eley.

### REFERENCES

BALDWIN, R. W. (1966) Tumour-specific immunity against spontaneous rat tumours. *Int. J. Cancer*, **1**, 257.

BALDWIN, R. W. (1979) Immunotherapy of cancer: Developing concepts and clinical progress. In *Ad. Med. Oncol. Res. Ed.*, **6**, p. 47.

BALDWIN, R. W. & EMBLETON, M. J. (1969) Immunology of spontaneously arising rat mammary adenocarcinomas. *Int. J. Cancer*, **4**, 430.

BALDWIN, R. W. & EMBLETON, M. J. (1974) Neoantigens on spontaneous and carcinogen-induced rat tumours defined by *in vitro* lymphocytotoxicity. *Int. J. Cancer*, **13**, 433.

BALDWIN, R. W. & PIMM, M. V. (1978) BCG in tumour immunotherapy. *Adv. Cancer Res.*, **28**, 91.

BARTLETT, G. L., ZBAR, B. & RAPP, H. J. (1972) Suppression of murine tumor growth by immune reaction to the Bacillus Calmette-Guérin strain of *Mycobacterium bovis*. *J. Natl Cancer Inst.*, **48** 245.

EMBLETON, M. J. & BALDWIN, R. W. (1980) Antigenic changes in chemical carcinogenesis. *Br. Med. Bull.*, **36**, 83.

GREAGER, J. A. & BALDWIN, R. W. (1978) Influence of immunotherapeutic agents on the progression of spontaneously arising rat mammary adenocarcinomas of varying immunogenicities. *Cancer Res.*, **38**, 69.

HAMMOND, W. G., FISHER, J. D. & ROLLEY, R. J. (1967) Tumor-specific transplantation immunity to spontaneous mouse tumours. *Surgery*, **62**, 124.

HEWITT, H. B. (1978) The choice of animal tumours for experimental studies of cancer therapy. *Adv. Cancer Res.*, **27**, 149.

HEWITT, H. B. (1979) A critical examination of the foundations of immunotherapy for cancer. *Clin. Radiol.*, **30**, 361.

HEWITT, H. B., BLAKE, E. R. & WALDER, A. S. (1976) A critique of the evidence for active host defence against cancer based on personal studies of 27 murine tumours of spontaneous origin. *Br. J. Cancer*, **33**, 241.

KLEIN, G. & KLEIN, E. (1977) Rejectability of virus-induced tumours and non-rejectability of spontaneous tumors: A lesson in contrasts. *Transpl. Proc.*, **9**, 1095.

MARTIN, D. S., STOLFI, R. L. & FUGMANN, R. A. (1977) Animal models for tumor immunotherapy: A commentary. *Cancer Immunol. Immunother.*, **2**, 77.

MILAS, L. & SCOTT, M. T. (1978) Antitumor activity of *Corynebacterium parvum*. *Adv. Cancer Res.*, **26**, 257.

MOORE, M., LAWRENCE, N. & NISBET, N. W. (1975) Tumour inhibition mediated by BCG in immunosuppressed rats. *Int. J. Cancer*, **15**, 897.

PIMM, M. V., COOK, A. J., HOPPER, D. G., DICKINSON, A. M. & BALDWIN, R. W. (1978) BCG treatment of transplanted rat tumours of spontaneous origin. *Int. J. Cancer*, **22**, 426.

PREHN, R. T. & MAIN, J. M. (1957) Immunity to methylcholanthrene-induced sarcomas. *J. Natl Cancer Inst.*, **18**, 769.

WILLMOTT, N., PIMM, M. V. & BALDWIN, R. W. (1979) *C. parvum* treatment of transplanted rat tumours of spontaneous origin. *Int. J. Cancer*, **24**, 323.

WRATHMELL, A. B. & ALEXANDER, P. (1976) Immunogenicity of a rat leukaemia of spontaneous origin. *Br. J. Cancer*, **33**, 181.

VASA-THOMAS, K. A., AMBROSE, K. R., BELLOMY, B. B. & COGGIN, J. H. (1977) Characterisation of immune responses to spontaneous hamster lymphomas. *J. Natl Cancer Inst.*, **58**, 1287.